# Involvement of ERK and Oxidative Stress in Airway Exposure to Cadmium Chloride Aggravates Airway Inflammation in Ovalbumin-Induced Asthmatic Mice

**DOI:** 10.3390/toxics12040235

**Published:** 2024-03-23

**Authors:** Chendong Wu, Xinyue Hu, Yuanyuan Jiang, Jiale Tang, Huan Ge, Shuanglinzi Deng, Xiaozhao Li, Juntao Feng

**Affiliations:** 1 Department of Respiratory Medicine, National Key Clinical Specialty, Branch of National Clinical Research Center for Respiratory Disease, Xiangya Hospital, Central South University, Changsha 410005, China; 2 National Clinical Research Center for Geriatric Disorders, Xiangya Hospital, Central South University, Changsha 410005, China; 3 Department of Nephrology, Xiangya Hospital, Central South University, Changsha 410005, China

**Keywords:** asthma, cadmium, ERK, oxidative stress, α-tocopherol

## Abstract

Inhalation represents a significant route of cadmium (Cd) exposure, which is associated with an elevated risk of lung diseases. This research study aims to evaluate the impact of repeated low-dose cadmium inhalation on exacerbating airway inflammation induced by ovalbumin (OVA) in asthma-afflicted mice. Mice were grouped into four categories: control (Ctrl), OVA, cadmium chloride (CdCl_2_), and OVA + cadmium chloride (OVA + CdCl_2_). Mice in the OVA group displayed increased airway mucus secretion and peribronchial and airway inflammation characterized by eosinophil cell infiltration, along with elevated levels of Th2 cytokines (IL-4, IL-5, IL-13) in bronchoalveolar lavage fluids (BALFs). These parameters were further exacerbated in the OVA + CdCl_2_ group. Additionally, the OVA + CdCl_2_ group exhibited higher levels of the oxidative stress marker malondialdehyde (MDA), greater activity of glutathione peroxidase (GSH-Px), and higher phosphorylation of extracellular regulated kinase (ERK) in lung tissue. Treatment with U0126 (an ERK inhibitor) and α-tocopherol (an antioxidant) in the OVA + CdCl_2_ group resulted in reduced peribronchial and airway inflammation as well as decreased airway mucus secretion. These findings indicate that CdCl_2_ exacerbates airway inflammation in OVA-induced allergic asthma mice following airway exposure. ERK and oxidative stress are integral to this process, and the inhibition of these pathways significantly alleviates the adverse effects of CdCl_2_ on asthma exacerbation.

## 1. Introduction

Allergic asthma constitutes a widespread chronic inflammatory airway disease characterized by airway hyper-responsiveness, mucus secretion, and airway remodeling [1]. The development of asthma is influenced by an interplay between genetic predispositions and environmental factors, with abnormal environmental factors such as air pollution and allergens playing a substantial role in its onset [2].

Cadmium (Cd), a heavy metal, is recognized as a toxic substance. The sources of Cd include the smelting of copper and nickel as well as the combustion of fossil fuels like coal and petroleum [3]. Additionally, electronic factories and electronic waste further contribute to Cd contamination [4]. The primary routes of human exposure are inhalation and ingestion, leading to the accumulation of Cd in vital organs [5,6], including the kidneys, lungs, and intestines [7]. Exposure to Cd can result in various types of systemic damage, including liver and kidney dysfunction, pulmonary edema, and reproductive toxicity [4,8,9]. Moreover, Cd has been identified as a carcinogen, with occupational or environmental exposure being associated with lung, breast, bladder, and other types of cancers [10,11,12].

Mounting evidence suggests an association between Cd exposure and asthma development. A case–control study conducted in Wuhan, China, revealed a positive correlation between elevated urine Cd levels and a higher risk of asthma among adult patients [13]. Similarly, Yang et al. conducted a cross-sectional study that showed a significant positive link between Cd levels in peripheral blood and asthma prevalence in adults using data from the National Health and Nutrition Examination Survey (NHANES) from 2007 to 2012. Additionally, they also observed that higher blood Cd levels were associated with a decreased forced expiratory volume in one second (FEV1) to forced vital capacity (FVC) ratio [14]. Data from the Korean National Health and Nutrition Examination Survey also demonstrated a relationship between blood Cd levels and asthma prevalence, as reported through participant self-reports and peripheral blood testing [15]. Wang et al. reported that early-life Cd exposure through the gastrointestinal tract exacerbated airway hyper-responsiveness, goblet cell hyperplasia, and mucus secretion in a mouse asthma model, although it did not increase the expression of Th2 cytokines in the lungs of asthmatic mice [16]. These studies suggest a potential connection between Cd exposure and both increased asthma incidence and symptom aggravation. As Cd is a significant toxic component in atmospheric fine particles capable of inducing pulmonary inflammation, the inhalation of atmospheric fine particles and air pollutants remains the primary route of Cd exposure for most individuals [5]. However, there is a paucity of research on the connections between airway Cd exposure, asthma pathogenesis, and the mechanisms involved.

Recent studies suggest that ERK signaling may play a significant role in the impact of Cd on pulmonary inflammation. For instance, Larson-Casey et al. observed that Cd exacerbates lower respiratory tract infections in mice by triggering ERK activation [17]. Additionally, Cao et al. revealed that Cd induces ERK pathway activation, leading to the apoptosis of human bronchial epithelial cells (BEAS-2B) [18]. Conversely, other investigations have highlighted the pivotal role of oxidative stress in Cd-induced lung injury in mice [19]. Although ERK and oxidative stress are implicated in asthma, their involvement in Cd’s effects on asthma remains unclear [20,21,22].

In this research, we employed an ovalbumin (OVA)-induced asthma mouse model to explore the effects of cadmium (Cd) on asthma pathogenesis via tracheal instillation of cadmium chloride (CdCl_2_). Our results demonstrated that exposure to CdCl_2_ intensifies airway inflammation and enhances eosinophil infiltration and the production of Th2 cytokines in asthma-afflicted mice. Mechanistically, our findings indicate the involvement of ERK activation and oxidative stress in exacerbating airway inflammation in asthma mice, which was substantiated by the efficacious use of inhibitors targeting ERK and oxidative stress pathways.

## 2. Materials and Methods

### 2.1. Animals Model

**Figure 1:**
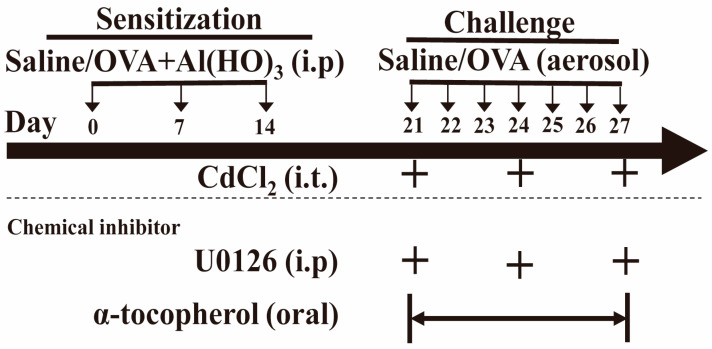
This figure illustrates a schematic of the experimental protocol. Notes: ovalbumin (OVA), aluminum hydroxide (Al(HO)_3_), cadmium chloride (CdCl_2_), U0126 (ERK inhibitor), α-tocopherol (antioxidant radical scavenger), intraperitoneally injected (i.p), intratracheal instillation (i.t). The bidirectional arrows indicate a continuous seven-day span from Day 21 to Day 27.

Male wild-type C57BL/6 mice, aged 6–8 weeks and weighing 18–20 g, were acclimatized at the Experimental Animal Center of Central South University in Changsha, Hunan, China, for one week before the experiment. The mice were kept under controlled conditions, including a temperature range of 21–25 °C, access to food and water ad libitum, and a 12 h light–dark cycle. An allergic asthma model was then induced in the mice. Specifically, on days 0, 7, and 14, the mice were intraperitoneally injected with 0.2 mL of sterile saline containing ovalbumin (OVA, 50 µg, Sigma, St. Louis, MO, USA) and aluminum hydroxide (2 mg, Sigma, St. Louis, MO, USA) for sensitization. Subsequently, from days 21 to 27, the mice were exposed daily to 5% OVA aerosol (w/v) for 30 min [23]. To investigate the impact of cadmium chloride (CdCl_2_, Sigma, USA) on asthma in mice, on days 21, 24, and 27, mice in the CdCl_2_ group and OVA + CdCl_2_ group received intratracheal instillation (i.t.) of 0.2 µg CdCl_2_ (50 µL in saline), whereas the control group and OVA group received saline instillation [24]. Finally, all mice were randomly allocated into four groups (*n* = 6): control group (Ctrl), OVA group, CdCl_2_ group, and OVA + CdCl_2_ group. On day 28, the mice were euthanized, and bronchoalveolar lavage fluid (BALF) and tissues were collected for further analysis, as outlined in the experimental protocol depicted in Figure 1.

### 2.2. Chemical Inhibitor Administration

In the experimental setup, the OVA + CdCl_2_ mouse model group were treated concomitantly with the ERK inhibitor U0126 and the antioxidant radical scavenger α-tocopherol. On days 21, 24, and 27, the mice received an intraperitoneal injection daily of 10 mg/kg U0126 (MedChem Express, Monmouth Junction, NJ, USA) dissolved in sterile enzyme-free water with 2% DMSO. Alternatively, α-tocopherol (15 IU/kg) (Sigma Aldrich, St. Louis, MO, USA), dissolved in 50% ethanol with a total volume of 10 μL, was administered orally twice a day from day 21 to day 27. These treatments were administered one hour prior to each instance of intratracheal instillation of CdCl_2_ or before the nebulization of ovalbumin (OVA) [25,26,27,28], as outlined in the experimental protocol depicted in Figure 1.

### 2.3. Bronchoalveolar Lavage Fluid (BALF) and Histopathological Collection

Upon euthanizing the mice, bronchoalveolar lavage (BAL) fluid was collected according to established protocols. The skin and tissues surrounding the neck region of the mice were dissected to expose the trachea. A syringe needle with a volume of 1 mL was inserted into the trachea for lavage, which was performed twice using 1 mL of phosphate-buffered saline (PBS). The collected BAL fluid was then centrifuged at 4 °C and 1500 rpm for 10 min, and the supernatant was utilized for ELISA analysis to identify inflammatory markers. The cellular pellets derived from the centrifugation were resuspended in PBS, and the proportion of eosinophils was determined using flow cytometry. Eosinophils were identified as CD45 + SiglecF + CD11c- [29]. The concentrations of IL-4, IL-5, and IL-13 in the BAL fluid were quantified using commercial ELISA kits (RENJIEBIO, Shanghai, China) following the provided protocols.

Left lung tissue excised from the mice following perfusion with PBS underwent fixation in 4% paraformaldehyde (ECOTOP, Guangzhou, China) for 48 h. Subsequently, the samples were embedded in paraffin, sectioned at 5 µm thickness, and stained with hematoxylin and eosin (H&E; ECOTOP, Guangzhou, China) and periodic acid–Schiff (PAS; Servicebio, Wuhan, China) for comprehensive analysis. We calculated the inflammation score to quantify lung damage, as previously described. Briefly, perivascular and peribronchiolar inflammation scores were assigned as follows: a score of 0 indicated normal tissue; 1 indicated the presence of a few cells; 2 corresponded to a single-cell layer of inflammatory cells; 3 corresponded to a two-to-four-cell layer; and a score of 4 indicated a layer of inflammatory cells exceeding four cells in thickness. To assess PAS-positive goblet cells within the airways, we adopted a relative scoring system: a score of 0 indicated less than 5% positive cells; 1 displayed 5–25% positivity; 2 indicated 25–50% positivity; 3 denoted 50–75% positivity; and 4 corresponded to more than 75% positivity [30].

### 2.4. MDA and GSH-Px Analysis

Following the collection of bronchoalveolar lavage fluid (BALF), right lung tissue was excised to measure the levels of malondialdehyde (MDA) and glutathione peroxidase (GSH-Px) activity, while the residual lung tissue was utilized for immunoblotting analysis. Measurements were conducted using commercial kits following the protocols provided by the manufacturer (Nanjing Jiancheng Bioengineering Institute, Nanjing, China).

### 2.5. Immunoblotting

The right lungs of the mice were homogenized with a tissue grinder (Tiangen, Beijing, China) and then incubated in RIPA lysis buffer (Beyotime, Shanghai, China) enriched with 1 mmol/L phenylmethanesulfonyl fluoride (PMSF) (MilliporeSigma, Billerica, MA, USA) and a protease inhibitor cocktail (Servicebio, Wuhan, China) for 30 min. Following homogenization, the samples were centrifuged at 12,000× *g* for 15 min at 4 °C, and protein levels in the supernatants were determined using a BCA protein assay kit (Beyotime, Shanghai, China). An equal quantity of protein (20 µg) from each sample was subjected to 10% SDS-PAGE, and thereafter, the proteins were transferred to polyvinylidene fluoride (PVDF) membranes. The membranes were incubated in 5% non-fat dry milk dissolved in Tris-buffered saline with 0.1% Tween-20 (TBST) to block non-specific binding sites for one hour. Following this, the membranes were incubated overnight at 4 °C with anti-phospho-ERK and total ERK primary antibodies (1:1000, Cell Signaling Technology, Danvers, MA, USA) as well as with anti-beta Actin primary antibodies (1:10,000, HUABIO, Hangzhou, China). Subsequently, the membranes underwent incubation with a secondary antibody at room temperature for 1 h. Ultimately, the blots were visualized using a chemiluminescence detection system (Advansta, Menlo Park, CA, USA).

### 2.6. Statistical Analysis

Statistical analyses were conducted using GraphPad Prism version 8.2.1 (GraphPad Software, La Jolla, CA, USA). One-way analysis of variance (ANOVA) with Tukey’s multiple comparisons test or two-way ANOVA was employed as suitable for the analysis. The data are reported as means ± standard error of the mean (SEM). The threshold for statistical significance was set at *p* < 0.05.

## 3. Results

### 3.1. Effects of CdCl_2_ Exposure on the Allergic Asthma Model

**Figure 2:**
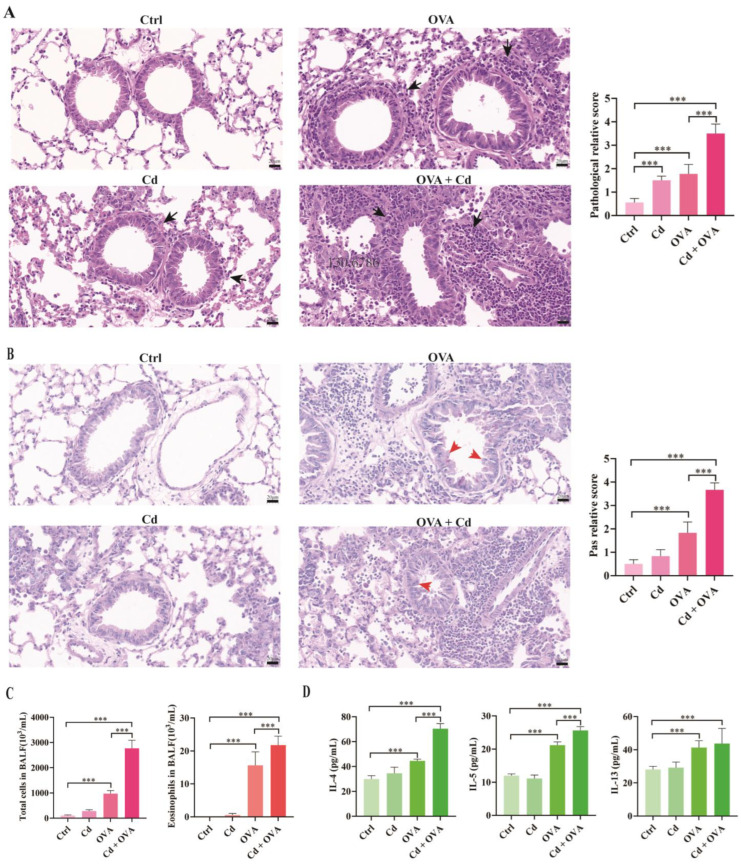
Effects of CdCl_2_ exposure on an allergic asthma mouse model (*n* = 6). (**A**) Lung tissue stained with hematoxylin and eosin (H&E) (**left**) and histological inflammation scores (**right**). Bar = 20 μm, magnification 400×. Black arrows indicate inflammatory cells. (**B**) Lung tissue stained with periodic acid–Schiff (PAS) (**left**) and PAS staining scores (**right**). Bar = 20 μm, magnification 400×. Red arrows indicate mucus. (**C**) Total and eosinophil cell counts in bronchoalveolar lavage fluids (BALFs) assessed by flow cytometry. (**D**) Cytokines (IL-4, IL-5, and IL-13) in BALFs measured by ELISA. Statistical significance is denoted by *** *p* < 0.001, as determined through one-way ANOVA. Control (Ctrl), ovalbumin (OVA), cadmium chloride (Cd).

Firstly, we examined pathological alterations in the lung tissues. Our findings revealed a notable rise in peribronchial inflammatory cell infiltration and elevated inflammation scores in the OVA group, CdCl_2_ group, and OVA + CdCl_2_ group compared to the control group. Moreover, the OVA + CdCl_2_ group exhibited heightened peribronchial inflammatory cell infiltration and inflammation scores in comparison to the OVA group (Figure 2A).

Periodic acid–Schiff (PAS) staining was employed to assess the level of mucus production by airway goblet cells and showed a significant elevation in PAS staining scores in the OVA group and the OVA + CdCl_2_ group compared to the control group. Additionally, the OVA + CdCl_2_ group exhibited further increases in PAS staining scores versus the OVA group, while exposure to CdCl_2_ alone did not affect PAS scores (Figure 2B). These findings suggest that airway exposure to CdCl_2_ can exacerbate mucus secretion in asthmatic mice.

Subsequently, we evaluated inflammatory cell profile changes in the BALFs of mice across different groups. As anticipated, a substantial increase in total cells and eosinophils was observed in the BALF of the OVA group. Exposure to CdCl_2_ via the airways exacerbated the levels of total cells and eosinophils in the BALF of the OVA + CdCl_2_ group. However, compared to the control group, CdCl_2_ exposure alone did not influence the levels of total cells and eosinophils (Figure 2C).

Moreover, an in-depth analysis was performed on the concentrations of Th2-related cytokines (IL-4, IL-5, IL-13) in the BALFs of mice. As depicted in Figure 2D, these cytokines exhibited elevation in both the OVA group and the OVA + CdCl_2_ group; notably, airway exposure to CdCl_2_ magnified the expression levels of IL-4 and IL-5, suggesting a synergistic effect of CdCl_2_ on the expression of IL-4 and IL-5 in asthmatic mice.

### 3.2. Airway Exposure to CdCl_2_ Enhances ERK Phosphorylation and Oxidative Stress in OVA-Induced Mice

**Figure 3:**
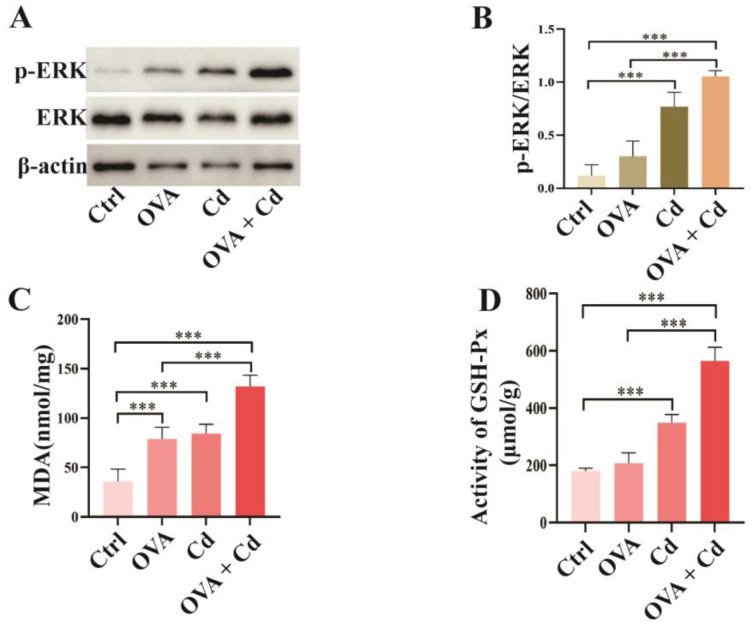
Airway exposure to CdCl_2_ enhances ERK phosphorylation and oxidative stress in OVA-induced mice. (**A**,**B**) The effect of CdCl_2_ exposure on phosphorylation of ERK in mouse lung tissues was investigated and quantitatively analyzed using Western blot (*n* = 4). (**C**,**D**) Oxidative stress was measured by the level of malondialdehyde (MDA) and the activity of glutathione peroxidase (GSH-Px) in mouse lung tissues (*n* = 6). Statistical significance is denoted by *** *p* < 0.001, as determined through one-way ANOVA. Control (Ctrl), ovalbumin (OVA), cadmium chloride (Cd), malondialdehyde (MDA), glutathione peroxidase (GSH-Px).

Evidence indicates that ERK and oxidative stress are involved in CdCl_2_-induced inflammation in the lungs of humans and mice [4,17,19]. This suggests that these two factors may intensify the exacerbation of asthma induced by CdCl_2_. To explore this, we assessed the ERK signaling pathway by measuring the phosphorylation level of ERK and the oxidative stress level in lung tissues. Compared to the control group, the phosphorylation level of ERK was significantly increased in both the CdCl_2_ group and the OVA + CdCl_2_ group. As expected, CdCl_2_ exposure further elevated the phosphorylation level of ERK in the OVA-induced asthma mice (OVA + CdCl_2_ group) compared to the OVA group. These findings suggest that the ERK pathway may play a crucial role in exacerbating asthma induced by CdCl_2_ (Figure 3A,B; Supplementary Figure S1A).

Malondialdehyde (MDA) levels and glutathione peroxidase (GSH-Px) activity serve as oxidative stress markers. In comparison with the control group, MDA levels were elevated in the lung tissues of mice in both the OVA group and the CdCl_2_-exposed group. CdCl_2_ exposure further increased the MDA levels in the OVA-induced asthma mice (OVA + CdCl_2_ group). The effect of CdCl_2_ exposure on GSH-Px activity mirrored that on MDA levels, except in the OVA group, indicating a synergistic impact of CdCl_2_ on the expression of MDA and GSH-Px in mouse lung tissues induced by OVA (Figure 3C,D).

### 3.3. Effect of U0126 and α-Tocopherol Treatment on the OVA + CdCl_2_ Mouse Model

**Figure 4:**
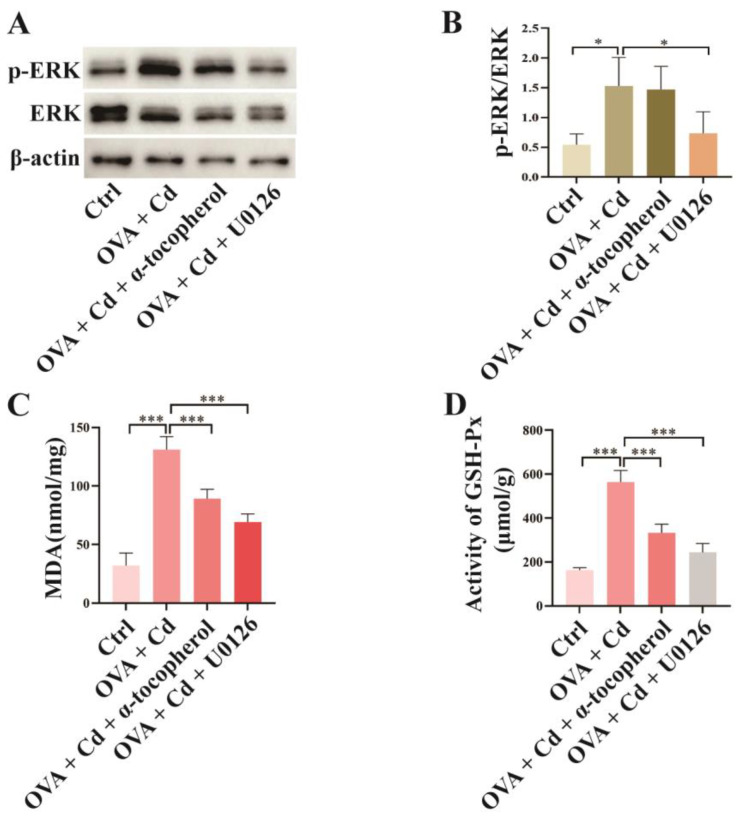
Effect of U0126 and α-tocopherol treatment on OVA + CdCl_2_ mice. OVA + CdCl_2_ mice were intraperitoneally injected with U0126 (10 mg/kg) or were orally fed α-tocopherol (15 IU/kg). (**A**,**B**) The phosphorylation of ERK in mouse lung tissues was investigated and quantitatively analyzed using Western blot (*n* = 4). (**C**,**D**) Oxidative stress was measured by the level of malondialdehyde (MDA) and the activity of glutathione peroxidase (GSH-Px) in mouse lung tissues (*n* = 6). Statistical significance is denoted by * *p* < 0.05 and *** *p* < 0.001, as determined through one-way ANOVA. Control (Ctrl), ovalbumin (OVA), cadmium chloride (Cd), malondialdehyde (MDA), glutathione peroxidase (GSH-Px), U0126 (ERK inhibitor), α-tocopherol (antioxidant radical scavenger).

To investigate the roles of ERK phosphorylation and oxidative stress in the exacerbation of lung inflammation caused by CdCl_2_ exposure in OVA-induced asthma mice, we administered the ERK inhibitor U0126 and the antioxidant α-tocopherol to the mice in the OVA + CdCl_2_ group. Figure 4A,B demonstrate that U0126 treatment significantly decreased ERK phosphorylation levels in mouse lung tissue compared to the OVA + CdCl_2_ group, whereas α-tocopherol did not impact ERK phosphorylation (Supplementary Figure S1B). In terms of oxidative stress markers, the administration of α-tocopherol and U0126 reduced MDA levels in mouse lung tissue compared to the OVA + CdCl_2_ group (Figure 4C). Similar results were observed in the changes in GSH-Px activity in mouse lung tissue (Figure 4D).

### 3.4. Role of ERK and Oxidative Stress in CdCl_2_-Exacerbated Asthma in Mice

**Figure 5:**
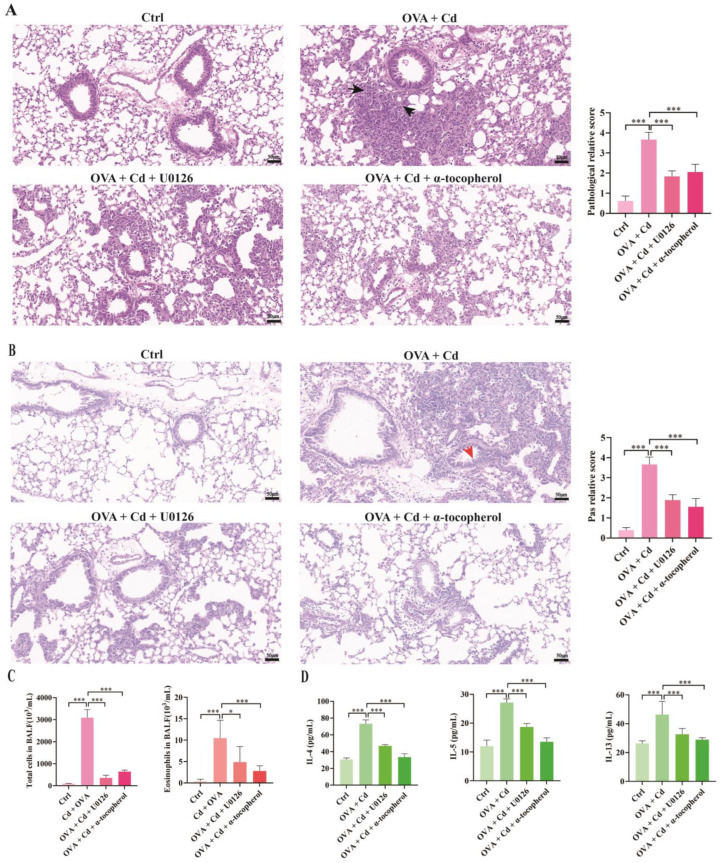
Role of ERK and oxidative stress in exacerbating the effects of CdCl_2_ on OVA-induced mice. OVA + CdCl_2_ mice were intraperitoneally injected with U0126 (10 mg/kg) or orally fed α-tocopherol (15 IU/kg). (**A**) Lung tissue stained with hematoxylin and eosin (H&E) (**left**) and histological inflammation scores (**right**) (*n* = 6). Bar = 50 μm, magnification 200×. Black arrows indicate inflammatory cells. (**B**) Lung tissue stained with periodic acid–Schiff (PAS) (**left**) and PAS staining scores (**right**) (*n* = 6). Bar = 50 μm, magnification 200×. Red arrows indicate mucus. (**C**) Total and eosinophil cell counts in bronchoalveolar lavage fluids (BALFs) were assessed by flow cytometry (*n* = 5). (**D**) Cytokines (IL-4, IL-5, and IL-13) in BALFs were measured by ELISA (*n* = 6). Statistical significance is denoted by * *p* < 0.05, and *** *p* < 0.001, as determined through one-way ANOVA. Control (Ctrl), ovalbumin (OVA), cadmium chloride (Cd), U0126 (ERK inhibitor), α-tocopherol (antioxidant radical scavenger).

Following the selective targeting of ERK and oxidative stress using a chemical inhibitor, we performed histopathological and biochemical assays to explore the role of these pathways in exacerbating inflammation in OVA-induced asthma mice treated with CdCl_2_. Figure 5A depicts that the histopathological inflammation scores of lung tissues from the mice were assessed to determine the impact of ERK phosphorylation and oxidative stress on airway inflammation in asthmatic mice exposed to CdCl_2_. Based on histological images and inflammation scores, treatment of asthmatic mice exposed to CdCl_2_ with the ERK inhibitor U0126 revealed a significant decrease in inflammation scores in the peribronchial area of lung tissue. Similarly, treatment with α-tocopherol also decreased inflammation scores in the peribronchial area of lung tissue.

Similarly, PAS staining served to determine the extent of mucus secretion in pulmonary airway goblet cells. As shown in Figure 5B, treatment with U0126 alleviated airway mucus secretion in asthma mice treated with CdCl_2_. Similarly, treatment with α-tocopherol also alleviated airway mucus secretion in asthma mice treated with CdCl_2_.

Next, we evaluated alterations in the total cell and eosinophil counts in bronchoalveolar lavage fluids (BALFs) collected from mouse airways. Figure 5C shows the effects of U0126 and α-tocopherol on airway inflammation and cell infiltration in OVA + CdCl_2_ mice. Administration of U0126 reduced the total cell count and eosinophil count in the BALF of asthma mice. On the other hand, the administration of α-tocopherol also reduced the total cell count and eosinophil count in mouse BALFs.

The periodic acid–Schiff (PAS) staining technique was employed to assess the level of mucus secretion in goblet cells of the pulmonary airways. Figure 5B demonstrates that treatment with U0126 mitigated airway mucus secretion in CdCl_2_-treated asthma mice. Similarly, treatment with α-tocopherol also diminished airway mucus secretion in asthma mice exposed to CdCl_2_. Subsequently, our investigation focused on the variations in the total cell count and eosinophil count in the bronchoalveolar lavage fluid (BALF) collected from mice airways. In Figure 5C, the impact of U0126 and α-tocopherol on airway inflammation and cell infiltration in OVA + CdCl_2_ mice is presented. U0126 administration attenuated total cell and eosinophil counts in the BALF of asthma mice. Similarly, administration of α-tocopherol resulted in a reduction in the total cell and eosinophil counts in the BALF.

Finally, the expression levels of Th2 cytokines in the bronchoalveolar lavage fluid (BALF) of mice were assessed. Figure 5D demonstrates that treating OVA + CdCl_2_ mice with U0126 resulted in decreased levels of IL-4, IL-5, and IL-13 Th2 cytokine levels in the BALF. Correspondingly, α-tocopherol administration also reduced the levels of these cytokines. The aforementioned findings indicate that ERK signaling and oxidative stress are implicated in the exacerbation of inflammation in mice with ovalbumin (OVA)-induced asthma following exposure to CdCl_2_.

## 4. Discussion

This study aimed to examine the effects of CdCl_2_ exposure via the respiratory tract on an OVA-induced asthma mouse model. The findings revealed that inhalation of CdCl_2_ intensified airway and lung inflammation in asthma mice, characterized by increasing eosinophil infiltration in the airways and promoting the secretion of Th2 cell cytokines in the BALF. Furthermore, this study highlighted the significant involvement of ERK and oxidative stress in these processes.

Previous research has touched upon the association between CdCl_2_ exposure and asthma, evidenced by clinical case studies that confirm this association [13,14,15]. At the animal level, Wang et al. investigated the impact of oral CdCl_2_ intake on asthma [16]. CdCl_2_ is recognized as a crucial toxic component in atmospheric particulate matter and air pollutants, leading to widespread human exposure through inhalation [5]. Nonetheless, a research gap remains concerning the specific impact of respiratory exposure to CdCl_2_ on asthma. Hence, this study distinguishes itself by concentrating on respiratory exposure in mice. Given the potential exacerbation of lung diseases through low-level Cd inhalation, it becomes imperative to evaluate the adverse consequences of such exposure in high-risk populations susceptible to toxic injury. The main aim of this study was to determine whether low-dose Cd inhalation exacerbates OVA-induced airway and lung tissue inflammation in asthmatic mice. While intratracheal instillation is commonly used in many studies [24], our approach involved exposing mice to Cd through the airways during antigen challenge, providing a more realistic simulation of Cd inhalation’s impact on asthma [26]. The results demonstrated that repeated airway exposure to low-dose CdCl_2_ induced airway and lung tissue inflammation, exacerbating OVA-induced inflammation in mice.

The disparity between our study and the research conducted by Wang et al. lies in the methods of CdCl_2_ exposure [16]. Our findings indicate that inhalation exposure to CdCl_2_ through the airway elicited an upsurge in total cell count, eosinophil count, as well as an elevation in Th2 cytokines in the BALF of mice with asthma. In contrast, Wang et al. reported no significant increase in Th2 cytokines or eosinophils following oral administration of CdCl_2_ [16]. These results imply that exposure to CdCl_2_ through different routes can lead to varying levels of eosinophils and Th2 cytokines in the airways of mice with asthma.

It is important to note that our findings indicate no significant increase in total cells or eosinophils in the bronchoalveolar lavage fluid (BALF) of mice in the CdCl_2_ intervention group, which aligns with the trends reported by Kim, M. S. et al., in whose study mice received an intratracheal instillation of 0.2 μg CdCl_2_ every three days for a total of four treatments [24]. In contrast, research by Wang et al. discovered that after daily inhalation of 10 mg/L CdCl_2_ via nebulization for four hours over six months, mice exhibited a significant increase in total cell numbers in the BALF compared to the control group, though changes in eosinophil levels were not reported [5]. Another study, involving daily intratracheal administration of 1 mg/kg body weight CdCl_2_, demonstrated a time-dependent rise in inflammatory cells within the BALF from day 7 to 21 post treatment. Notably, a significant escalation in eosinophil counts was observed on day 21 compared to controls [31]. These results suggest that the method, timing, and duration of CdCl_2_ administration distinctly influence the concentration of inflammatory cells in the respiratory tract of mice. However, to date, there has been no report on whether CdCl_2_ exposure via the airways exacerbates Th2-type inflammatory responses in asthmatic mice.

Current research on the correlation between Cd and asthma primarily focuses on clinical observations. Studies have discovered that Cd exposure increases the incidence of asthma and may exacerbate symptoms. However, the underlying mechanisms remain unclear [3,13,15]. Wang et al. found that oral administration of CdCl_2_ to mice exacerbated airway hyper-reactivity, goblet cell hyperplasia, and mucus secretion, but did not detail the specific mechanisms [16]. Cd is mainly inhaled through the respiratory tract and can be deposited in the airways, damaging the mucosal barrier. This exposure inhibits macrophage, dendritic cell, and natural killer cell-mediated innate immune responses, promoting inflammatory cell infiltration and inflammatory mediator release. These processes are critical in the inflammatory response of the airways, a key factor in the onset of asthma [32,33,34].

The ERK pathway plays a pivotal role in the inflammation induced by CdCl_2_ in mouse lung tissue. Exposure to CdCl_2_ initiates ERK activation in mouse lung tissue, which subsequently orchestrates the degradation of peroxisome proliferator-activated receptor γ (PPARγ) in macrophages, thereby exacerbating pulmonary infection in mice [17]. Moreover, CdCl_2_ activation of the ERK pathway leads to apoptosis in human bronchial epithelial cells (BEAS-2B) in vitro [18]. These findings suggest that CdCl_2_ may induce inflammation in mouse lung tissue via the ERK pathway. In vitro studies have demonstrated that IL-4 and IL-13 enhance the secretion of eotaxin from human airway smooth muscle cells, and the inhibition of the ERK pathway can alleviate IL-4 and IL-13-mediated eotaxin secretion. Notably, IL-4, IL-13, and eotaxin play a significant role in the eosinophilia characteristic of asthma [35]. Research by Devi et al. illustrates that Tridax procumbens, a traditionally used medicinal plant known for its anti-inflammatory properties, can suppress ERK pathway activity and mitigate airway inflammation in a murine asthma model [36]. Similarly, Hyun Min Ko has arrived at comparable conclusions [37]. However, the role of ERK in the exacerbation of inflammation following CdCl_2_ airway exposure in asthmatic mice remains unclear, as does the potential for ERK pathway inhibition to alleviate this inflammation. Our research further reveals that the ERK pathway plays a crucial role in the intensification of asthma induced by CdCl_2_. We observed that co-exposure to CdCl_2_ and ovalbumin (OVA) leads to a synergistic increase in ERK phosphorylation. Conversely, administration of the ERK-specific inhibitor U0126 mitigates peribronchial and airway inflammation as well as mucus production in the airways of mice exposed to both OVA and CdCl_2_. In conclusion, our findings highlight the pivotal role of ERK in the exacerbation of airway inflammation triggered by CdCl_2_ in mice with asthma.

Previous research indicates that Cd exposure induces oxidative stress in murine lung tissue, enhances inflammatory cell infiltration, and increases pro-inflammatory cytokine levels, with suppression of oxidative stress leading to reduced pulmonary inflammation [19,32]. α-tocopherol, a form of vitamin E and a fat-soluble vitamin, has been shown to diminish the generation of reactive oxygen species [38]. As such, α-tocopherol plays a vital role in inflammatory diseases. For instance, Wallert observed that α-tocopherol significantly reduces inflammation and oxidative stress levels in a mouse model of myocardial ischemia–reperfusion injury [39]. Similarly, in a murine model of lipopolysaccharide (LPS)-induced acute lung injury (ALI), α-tocopherol lowered the expression of pro-inflammatory cytokines and increased the expression of the antioxidant molecules superoxide dismutase (SOD)1/2 and glutathione peroxidase (GSH-Px) [40]. Consequently, α-tocopherol has demonstrated potential therapeutic effects on inflammation and oxidative damage. However, its efficacy in alleviating inflammation aggravated by CdCl_2_ airway exposure in asthmatic mice remains unclear. Moreover, our findings reveal that oxidative stress significantly contributes to the worsening of airway inflammation in asthmatic mice exposed to CdCl_2_ through the airways. Firstly, exposure to CdCl_2_ resulted in higher levels of oxidative stress in the lung tissue of mice, as evidenced by increased MDA levels and increased GSH-Px activity. Additionally, CdCl_2_ exposure synergistically heightened oxidative stress levels in the lung tissue of asthmatic mice. The administration of the antioxidant α-tocopherol was found to mitigate oxidative stress, thereby reducing the exacerbation of peribronchial and airway inflammation, as well as the secretion of airway mucus. These results underscore that oxidative stress contributes to the exacerbation of airway inflammation in asthmatic mice exposed to CdCl_2_.

It is noteworthy that we observed a reduction in oxidative stress levels in the OVA + CdCl_2_ mouse model following ERK phosphorylation inhibition. Nonetheless, inhibiting oxidative stress did not influence ERK phosphorylation. These findings imply that the pathway of ERK phosphorylation may act as one of the upstream regulators of oxidative stress in the OVA + CdCl_2_ mouse model. While the precise molecular mechanisms through which ERK induces oxidative stress remain unclear, Wang et al. reported that inhibiting ERK-mediated oxidative stress successfully ameliorated lung inflammation in an acute respiratory distress syndrome (ARDS) mouse model, which is consistent with our observations [41]. Therefore, it seems that the ERK–oxidative stress pathway may contribute to the inflammatory response, meriting rigorous further investigation to clarify this relationship.

This study’s limitations include the need for subsequent research to investigate the implications of varying administration routes and schedules of CdCl_2_ on the progression of airway inflammation in asthmatic mice. In addition, the biomarkers used to evaluate oxidative stress should be broadened to enable a more comprehensive assessment. Moreover, gaining a thorough and integrated understanding of the role of ERK signaling and oxidative stress in exacerbating airway inflammation induced by CdCl_2_ in these mice is essential for elucidating the complex underlying mechanisms.

## 5. Conclusions

CdCl_2_ exposure through the airways significantly impacts various pathological and physiological aspects in mice with asthma, including increased peribronchial and airway inflammation and elevated levels of Th2-type cytokines in the airways. Moreover, exposure to CdCl_2_ via the airways enhances ERK phosphorylation and oxidative stress in the lung tissue of asthma-afflicted mice. This study offers a novel perspective on how the heavy metal Cd exacerbates inflammation in asthma and suggests that targeting the ERK and oxidative stress pathways may be an effective strategy for attenuating Cd-induced exacerbation of inflammation and worsening of asthma control. This inhibitory strategy has the potential to be extended to manage the worsening of asthma control caused by other heavy metals, such as lead [42]; however, comprehensive investigation is necessary to fully explore this possibility.

## Data Availability

The data presented in the article are available from the authors on request.

## Supplementary Materials

The following supporting information can be downloaded at: https://www.mdpi.com/article/10.3390/toxics12040235/s1, Figure S1: CdCl_2_-induced ERK phosphorylation and oxidative Stress in OVA-induced asthma mice: mitigation by U0126 and α-tocopherol.
