# Supplementary material for: Involvement of ERK and Oxidative Stress in Airway Exposure to Cadmium Chloride Aggravates Airway Inflammation in Ovalbumin-Induced Asthmatic Mice
Source: Toxics. 2024 Mar 23;12(4):235. doi: 10.3390/toxics12040235 (PMC11054730; doi:10.3390/toxics12040235)
Supplement: Supplementary file 1 [file toxics-12-00235-s001.zip › toxics-2908348-supplementary.pdf]

Supplementary Figure 1

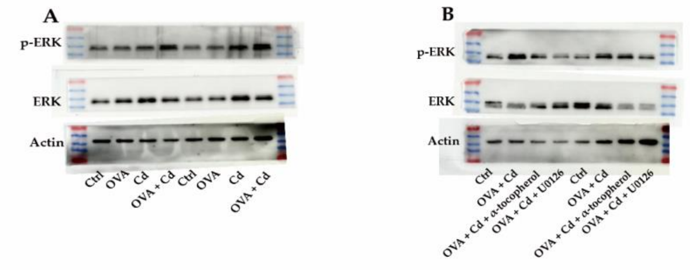

Supplementary Figure 1. $CdCl_2$-induced ERK phosphorylation and oxidative Stress in OVA-induced asthma mice: mitigation by U0126 and α-tocopherol. (**A**) Airway exposure to $CdCl_2$ enhances ERK phosphorylation in OVA-induced mice. (**B**) Effect of U0126 and α-tocopherol treatment on ERK phosphorylation in OVA + $CdCl_2$ mice.
